# Fibroblasts Promote Inflammation and Pain via IL-1α Induction of the Monocyte Chemoattractant Chemokine (C-C Motif) Ligand 2

**DOI:** 10.1016/j.ajpath.2017.11.007

**Published:** 2018-03

**Authors:** Hannah L. Paish, Nicholas S. Kalson, Graham R. Smith, Alicia del Carpio Pons, Thomas E. Baldock, Nicholas Smith, Katarzyna Swist-Szulik, David J. Weir, Michelle Bardgett, David J. Deehan, Derek A. Mann, Lee A. Borthwick

**Affiliations:** ∗ Fibrosis Research Group, Institute of Cellular Medicine, Newcastle University, Newcastle upon Tyne, United Kingdom; ‡ Bioinformatics Support Unit, Institute of Neuroscience, Newcastle University, Newcastle upon Tyne, United Kingdom; § Wellcome Centre for Mitochondrial Research, Institute of Neuroscience, Newcastle University, Newcastle upon Tyne, United Kingdom; † Musculoskeletal Unit, Freeman Hospital, Newcastle Hospitals, NHS Trust, Newcastle upon Tyne, United Kingdom

## Abstract

Fibroblasts persist within fibrotic scar tissue and exhibit considerable phenotypic and functional plasticity. Herein, we hypothesized that scar-associated fibroblasts may be a source of stress-induced inflammatory exacerbations and pain. To test this idea, we used a human model of surgery-induced fibrosis, total knee arthroplasty (TKA). Using a combination of tissue protein expression profiling and bioinformatics, we discovered that many months after TKA, the fibrotic joint exists in a state of unresolved chronic inflammation. Moreover, the infrapatellar fat pad, a soft tissue that becomes highly fibrotic in the post-TKA joint, expresses multiple inflammatory mediators, including the monocyte chemoattractant, chemokine (C-C motif) ligand (CCL) 2, and the innate immune trigger, IL-1α. Fibroblasts isolated from the post-TKA fibrotic infrapatellar fat pad express the IL-1 receptor and on exposure to IL-1α polarize to a highly inflammatory state that enables them to stimulate the recruitment of monocytes. Blockade of fibroblast CCL2 or its transcriptional regulator NF-κB prevented IL-1α–induced monocyte recruitment. Clinical investigations discovered that levels of patient-reported pain in the post-TKA joint correlated with concentrations of CCL2 in the joint tissue, such that the chemokine is effectively a pain biomarker in the TKA patient. We propose that an IL-1α–NF-κB–CCL2 signaling pathway, operating within scar-associated fibroblasts, may be therapeutically manipulated for alleviating inflammation and pain in fibrotic joints and other tissues.

Fibrosis is the excessive deposition of scar tissue arising within a tissue or joint after injury or trauma.^1^ Adhesions are fibrous bands that are generated after surgery; they form physical connections between tissues that are not normally directly connected, resulting in derangement and loss of function of the affected tissue or joint.^2^ Fibrous adhesions can also be associated with intermittent periods of acute inflammation, debilitating pain, and progressive degeneration of the affected tissue or joint.^3^ Therapeutic modulation of these acute exacerbations is limited by our current poor understanding of their underlying molecular triggers and mediators.

The cell type responsible for the deposition of fibrotic extracellular matrix is the fibroblast.^4^, ^5^ After tissue damage, resident pericytes, fibroblasts, or epithelial cells transdifferentiate to the fibroblast phenotype characterized by their expression of α-smooth muscle actin and abundant secretion of collagens.^6^ Resolution of wound healing is associated with the clearance of fibroblasts, whereas the maintenance and progression of fibrosis is associated with fibroblast proliferation and survival.^7^ Fibroblasts exhibit considerable phenotypic plasticity and may even reverse transdifferention or alternatively adopt an inactivated state characterized by reduced secretion of extracellular matrix proteins and an extended lifespan of several weeks.^8^, ^9^ An often underappreciated feature of fibroblasts is their ability to adopt an immune effector phenotype in response to stimulation with IL-1α/β or tumor necrosis factor-α.^10^, ^11^, ^12^, ^13^ We recently described how epithelial stress, via release of IL-1α, triggers the polarization of lung fibroblasts toward a proinflammatory phenotype characterized by their highly abundant secretion of IL-6, IL-8, chemokine (C-C motif) ligand (CCL) 2 (monocyte chemoattractant protein-1), and granulocyte-macrophage colony-stimulating factor.^14^, ^15^ Similar inflammatory cytokine release has also been described in hepatic stellate cells,^16^ cardiac fibroblasts,^17^ dermal fibroblasts,^18^ and fibroblasts isolated from the knee,^19^ suggesting the ability to adopt an immunomodulatory state may be a ubiquitous property of fibroblasts. Herein, we hypothesized that fibroblasts persisting within fibrotic microenvironments may be a source of stress-induced inflammatory exacerbations and pain.

To test this hypothesis, we used a human model of surgery-induced fibrosis, the post-surgical knee. Total knee arthroplasty (TKA) is a proven cost-effective treatment for painful end-stage knee osteoarthritis, in which the articulating joint surfaces are removed and replaced with new prosthetic-bearing surfaces.^20^, ^21^ After surgery, the TKA knee is characterized by a fibrotic process, the persistence of fibroblasts lasting months after surgery, and the deposition of vast quantities of fibrotic extracellular matrix.^22^, ^23^ Furthermore, approximately 20% of TKA patients report swelling and pain of their joint that can be more severe than that experienced before surgery.^24^, ^25^ By acquiring synovial fluid and tissue from patients undergoing primary TKA (nonfibrotic) and patients undergoing revision TKA (fibrotic), we were able to define the inflammatory profile of the fibrotic joint and a role for IL-1 receptor (IL-1R1), expressing inflammatory fibroblasts. Using a combination of bioinformatics and *in vitro* assays, we provide evidence that inflammatory fibroblasts regulate monocyte recruitment to fibrotic tissues via the release of CCL2 and demonstrate that CCL2 levels in the post-TKA joint correlate with reported pain.

## Materials and Methods

### Ethics

This study was performed in accordance with approval from the Newcastle and North Tyneside Local Regional Ethics Committee, and informed written consent was obtained from all patients (12/NE/0395). Samples used in this research were obtained from the Newcastle Biomedicine Biobank (Newcastle upon Tyne, UK; *http://www.ncl.ac.uk/nbb/collections*).

### Tissue Collection and Patient Stratification

All patients undergoing revision surgery for failed primary TKA were included in the study during a 2-year period, with infection being the only exclusion criterion. Synovial fluid, infrapatellar fat pad, and synovial membrane (suprapatellar pouch) were collected from patients undergoing either primary (*n* = 29) or revision (*n* = 33) TKA at the Freeman Hospital (Newcastle upon Tyne, UK) (Table 1).

**Table 1:** Patient Demographics

Variable	Sex, M:F ratio	Age in years, median (range)	BMI, median (range)	Time in months from primary to revision TKA, median (range)	Primary indication for surgery
Primary TKA (*n* = 29)	17:12	66 (44–81)	32 (22–47)	NA	Osteoarthritis (*n* = 28), rheumatoid disease (*n* = 1)
Revision TKA (*n* = 33)	16:17	71 (40–88)	33 (23–46)	106 (8–360)	Osteolysis and loose components (*n* = 11), clinical diagnosis of fibrosis with loss of movement (*n* = 11), primary laxity pattern with functional instability (*n* = 8), progression of osteoarthritis (previous patellofemoral resurfacing) (*n* = 3)

F, female; M, male; BMI, body mass index; NA, not applicable; TKA, total knee arthroplasty.

Revision TKA patients were stratified as those with osteolysis and loose components (*n* = 11), a clinical diagnosis of fibrosis with loss of movement (*n* = 11), primary laxity pattern with functional instability (*n* = 8, of which *n* = 6 had lost posterior cruciate ligament–required revision to a higher constraint prosthesis and *n* = 2 had midpoint laxity and the collateral ligaments were lax in full extension and underwent revision to a higher constraint implant), and progression of osteoarthritis (previous patellofemoral resurfacing) (*n* = 3). In the revision cohort, the primary knee replacements were as follows: 16 PFC (Sigma, St. Louis, MO), six Kinemax (Howmedica Osteonics Corporation, Mahwah, NJ), two Triathlon (Stryker, Kalamazoo, MI), one Genesis II (Smith and Nephew, London, UK), one Oxford unicompartmental (Zimmer, Warsaw, IN), one Vanguard (Biomet, Warsaw, IN), one AGC (Biomet), one IB2 (Zimmer), one Noiles (DePuy, Raynham, MA), and three primary patella-femoral resurfacing. Six revision patients with a total knee replacement *in situ* also had a patella-femoral resurfacing.

For the purposes of our study, we used a definition of primary joint fibrosis as previously described.^22^, ^26^ In all primary and revision TKA patients, local anesthetic was administered intraoperatively under direct vision (to a maximum dose of 100 mL of 0.2% ropivacaine). All patients were given routine postoperative analgesia, including regular paracetamol and a patient-controlled analgesia device. In addition, patients with restricted range of motion (fibrotic revisions) underwent continuous passive movement for 48 to 72 hours postoperatively.

Routine practice in our unit includes excision of the infrapatellar fat pad. All primary patients in the study had their infrapatellar fat pad removed. In the revision cohort, 24 had primary surgery performed in our unit and, therefore, had their infrapatellar fat pad removed in the primary procedure. The remaining nine revision patients had surgery elsewhere, and we do not know whether the infrapatellar fat pad was removed in their primary procedure. The infrapatellar fat pad tissue resected in revision surgery was from an anatomically matched site to the normal fat pad tissue found in the primary knee and resected in primary replacement surgery.

Synovial fluid was collected by aspirating fluid from the knee during surgery. The sample was sterile filtered (0.4 μm thick) and stored at −80°C. Representative tissue biopsy specimens from infrapatellar fat pad and synovial membrane were fixed in formalin and paraffin embedded. Serial tissue sections (5 μm thick) were cut and processed for staining, as described later. Tissue was homogenized in radioimmunoprecipitation assay buffer supplemented with protease and phosphatase inhibitors using a bead homogenizer (TissueLyserII; Qiagen, Hilden, Germany). Homogenized samples were normalized to 1 mg/mL total protein (protein concentration measured using a bicinchoninic acid protein assay; Pierce, Waltham, MA), as per manufacturer's instructions, and stored at −80°C

### Exclusion of Infection

Preoperative workup included C-reactive protein (all patients) and joint aspiration (7 of 33 revision patients). Joint aspiration was performed only when the treating clinician believed that infection needed to be excluded. C-reactive protein was <30 in all revision patients, and all preoperative joint aspiration samples were negative after extended culture. Infection was definitively ruled out in all revision patients by microbiological analysis of synovial fluid and of multiple tissue samples taken at revision surgery. All revision patients had negative extended culture of both intraoperative synovial fluid and tissue samples. A minimum of three and a maximum of six tissue samples were taken intraoperatively, according to prosthetic joint infection guidelines.^27^ As is standard practice, all orthopedic theater samples underwent extended enrichment culture for 10 days. However, a role for organisms, such as *Propionibacterium acnes*, that are challenging to culture, and may be an underrecognized cause of knee prosthetic joint infection, cannot be ruled out.^28^, ^29^

### Histological Analysis of Tissue Samples

Formal histology reports were available for 9 of 33 revision patients. All reports state that dense, hypocellular, heavily collagenized fibrous tissue foreign body giant cells containing small transparent, strongly birefringent foreign particles were identified. The appearances are those of extensive fibrosis together with a focal giant cell reaction to foreign material, a reaction to polyethylene debris from the prosthetic joint implant. There was no evidence of infection reported in any cases.

### Immunohistochemistry

Tissue sections (5 μm thick) were deparaffinized in xylene, rehydrated in graded alcohol, and incubated in 0.6% hydrogen peroxide/methanol for 15 minutes. Antigen retrieval was performed using 20 μg/mL Proteinase K (Sigma) in phosphate-buffered saline (PBS) at 37°C for 30 minutes. Blocking was performed using Avidin/Biotin Block (Vector Laboratories, Burlingame, CA), followed by porcine serum. Primary antibody incubation was performed overnight at 4°C using rabbit polyclonal primary antibody against IL-1R1 (Ab106278; Abcam, Cambridge, UK). Slides were washed with PBS and incubated with anti-rabbit biotinylated antibody (0.51 mg/L; Dako, Santa Clara, CA) in 1% porcine serum for 1 hour at room temperature. ABC tertiary (Vector Laboratories) was then applied to slides for 45 minutes at room temperature before application of 3,3′-diaminobenzidine mix (Vector Laboratories) for 5 minutes, followed by counterstaining with Mayer’s hematoxoylin (Sigma). Slides were dehydrated in graded ethanol and Clearene (Leica Biosystems, Wetzlar, Germany) before mounting using Pertex (CellPath Ltd., Newtown Powys, UK). Images were acquired using a Nikon Eclipse Upright Microscope (Nikon Instruments Europe BV, Amsterdam, the Netherlands). For CD68 image quantification, the mean number of positive cells in ×10 randomly selected high-powered fields (×20 magnification) was calculated using Nikon NIS elements image analysis software version 4.0 (Nikon Instruments Europe BV).

### Immunocytochemistry

Tissue sections (5 μm thick) were deparaffinized in xylene and rehydrated in graded alcohol. Antigen retrieval was performed with proteinase K (20 μg/mL in PBS) for 30 minutes at 37°C. Samples were blocked with 5% bovine serum albumin/PBS and incubated overnight at 4°C with primary antibodies diluted in 5% bovine serum albumin/PBS [IL-1R1, ab106278 (Abcam, Cambridge UK); IL-6, AF-206 at 10 μg/mL (R&D Systems, Minneapolis, MN); vimentin, M7020 at 62 μg/mL (Dako)]. Slides were washed with PBS-Tween (0.05%) and incubated with anti-rabbit tetrarhodamine isothiocyanate (T6778; Sigma-Aldrich) and anti-mouse fluorescein isothiocyanate (F2012; Sigma-Aldrich) diluted in 5% bovine serum albumin/PBS for 1 hour at room temperature. Slides were washed with PBS-Tween and mounted using mounting medium with DAPI (H1200; Vector Laboratories). Images were acquired on a Nikon A1R point scanning confocal microscope.

### Multiarray Protein Assays

Synovial fluid (primary, *n* = 21; revision, *n* = 24) and tissue homogenates from infrapatellar fat pad (primary, *n* = 28 patients; revision, *n* = 32 patients) and synovial membrane (primary, *n* = 29 patients; revision, *n* = 32 patients) were assessed for expression of 39 protein markers using a human V-Plex electrochemiluminescence detection kit (K15209D; MesoScaleDiscovery, Rockville, MD), as per manufacturer's instructions. Analysis of results was performed using the MSD Discovery Workbench software version 4.0 (MesoScaleDiscovery, Rockville, MD).

### Network Reconstruction

To identify potentially important intracellular signaling pathways/processes likely occurring in the revision TKA knee, we constructed a network using protein-protein interactions from InnateDB (*http://www.innatedb.com*). The differentially expressed markers and log(fold changes) were uploaded to the web server NetworkAnalyst (*http://www.networkanalyst.ca*),^30^ and the network was built with direct interactions only from the innate DB interactome.^31^ The resulting network contains 730 nodes and 921 edges and was visualized with Cytoscape.^32^ Statistics, such as degree (number of connections of a given node) and betweenness centrality (the number of paths between other nodes passing through a given node), were calculated. Modules (highly connected subnetworks) were found by a method based on the spreading rate of a random walk on the network. Significant gene ontology terms were identified using overrepresentation of the proteins in the network, or modules of the network, among gene sets annotated with these terms.

### Fibroblast Isolation

Infrapatellar fat pad isolated from patients undergoing primary and revision TKA was homogenized and digested in 10 mL of supplement-free medium [Dulbecco’s modified Eagle’s medium (DMEM); D5671; Sigma] containing 100 units/mL of collagenase (C6885; Sigma) for 60 minutes. Digested tissue was passed through a 100-μm filter, and the collagenase was neutralized with complete medium (DMEM plus 10% fetal calf serum, 100 μg/mL streptomycin, 100 U/mL penicillin, and 1% l-glutamate). Cells were pelleted and resuspended in complete medium before transferring into T-75 flasks. Cells were cultured at 37°C and 5% CO_2_ until >90% confluent. Cells were cryopreserved at passage number (P)0 or passaged and cryopreserved at P1. Mesenchymal phenotype was confirmed, as previously described.^19^ Fibroblasts were reanimated and used between P2 and P5 for all experiments in this study.

### Cell Treatments

Fibroblasts isolated from the infrapatellar fat pad of patients undergoing primary (*n* = 6 patients) or revision (*n* = 6 patients) TKA were serum starved for 24 hours before treatment with 0% fetal bovine serum (FBS) DMEM (control), 0% FBS DMEM + IL-1α (500 pg/mL), or 0% FBS DMEM + transforming growth factor (TGF)-β1 (3 ng/mL) for 24 hours. In some experiments, fibroblasts were pretreated with inhibitors [SP600125, SD1008, FR180204, SB203580, (5Z)-7-oxozeaenol, IL-1 receptor associated kinase 1/4 inhibitor 1 (all from Tocris, Minneapolis, MN) or Iκ B kinase (IKK)-2 inhibitor VIII (MerckMillipore, Billerica, MA)] for 1 hour before treatment with inhibitors + IL-1α (500 pg/mL). After 24 hours, the medium was collected, spun at 800 × *g* to clear, and stored at −80°C for analysis or use in transwell migration assays.

### Enzyme-Linked Immunosorbent Assay

IL-6, IL-8, and CCL2 protein levels in media samples from fibroblasts were quantified by ELISA (R&D Systems), as per manufacturer's instructions.

### Lactate Dehydrogenase Assay

Lactate dehydrogenase levels in media collected from fibroblasts were quantified using a Pierce LDH Cytotoxicity Assay Kit (Thermo Scientific, Waltham, MA), as per manufacturer's protocol.

### THP-1 Cell

THP-1 cells are an acute monocytic leukemia human cell line and were purchased from ATCC (TIB-202; ATCC, Manassas, VA). Cells were cultured in RPMI-1640 media (R0883; Sigma) supplemented with 10% FBS, 1% l-glutamine, 100 U/mL penicillin, and 100 μg/mL streptomycin. Cells were maintained at approximately 0.5 × 10^6^ cells/mL, and medium was changed twice weekly. THP-1 cells were used to provide a uniform monocyte population and ensure the only experimental variable in transwell migration assays was the composition of the conditioned media.

### Transwell Migration Assay

Media from fibroblasts treated with 0% FBS DMEM (control), 0% FBS DMEM + IL-1α (500 pg/mL), 0% FBS DMEM + TGF-β1 (3 ng/mL), or fibroblasts pretreated with inhibitors before treatment with 0% FBS DMEM + IL-1α were placed directly into the bottom of a 24-well plate. Recombinant CCL2 (100 pg/mL) in 0% FBS DMEM was used as a positive control, and 0% FBS DMEM was used as a negative control. THP-1 cells (50,000 in 100 μL 0% FBS DMEM) were added to a 6.5-mm, 5-μm pore transwell insert (Costar Corning, Corning, NY), and the insert was added to the 24-well plate. After 18 hours, migrated cells were imaged using a Nikon D5000 camera and quantified using ImageJ software version 1.41 (NIH, Bethesda, MD; *http://imagej.nih.gov/ij*). In some experiments, medium from fibroblasts treated with 0% FBS DMEM + IL-1α (500 pg/mL) was pretreated with neutralizing antibodies against CCL2 (4 μg/mL; AF-479; R&D Systems) and IL-6 (200 μg/mL; Ab6672; Abcam) for 1 hour before adding to the 24-well plate.

### Clustering and Stratification

In the clustering analysis, protein expression values were transformed according to log10(intensity + 2*δ), where δ is the smallest nonzero measured value. The measured markers were then sorted by fold change between primary and revision, and the top *n* values were used for clustering, where *n* was chosen so that (*n* + 1) is the first not significantly DE between the two groups or where this would lead to few proteins, *n* = 10. The distance measure between marker sets was the euclidean distance between the log-transformed expression, and Ward's method was used for hierarchical clustering. Heat maps were generated with the ComplexHeatmap package in R version 3.2.3 (The R Foundation for Statistical Computing, Vienna, Austria). For display, each row is Z transformed to equalize differences in overall expression. Principal component analysis was performed using the base R function princomp and all markers.

### Stratification with a Reduced Set of Markers

A reduced set of markers was selected with the R package glmnet (*http://www.jstatsoft.org/v33/i01*).^33^ The binomial family was used for the generalized linear model, with 10-fold cross validation to prevent overfitting. Data were weighted so that each class (primary or revision) had equal weight, despite different numbers of observations. In using the single marker CCL2 alone to classify primary/revision TKA patients, the model was fitted using the base R glm function and the receiver operating characteristic curve produced with the R package pROC.^34^ The median and CIs on the area under the curve were calculated with the ci.auc function.

### Statistical Analysis

Markers in synovial fluid, synovial membrane, and infrapatellar fat pat were grouped into those significantly elevated, those significantly decreased, and those not significantly different or undetectable in patients undergoing primary and revision TKA. Data are presented as a Venn diagram, graphically as change in median expression levels, as box-and-whiskers plots for individual markers, and in tabular form. Significance was calculated using *U*-test. Correlations between IL-1α levels and CCL2, IL-6, and IL-8 levels were tested using linear regression. *In vitro* data are presented as means ± SEM. Differences between treatment groups were analyzed using paired *t*-test (Graphpad Prism 6; GraphPad Software Inc., La Jolla, CA). Significance was set at *P* < 0.05.

## Results

### Chronic Inflammation Is a Common Feature of the Postsurgical Joint

**Figure 1:**
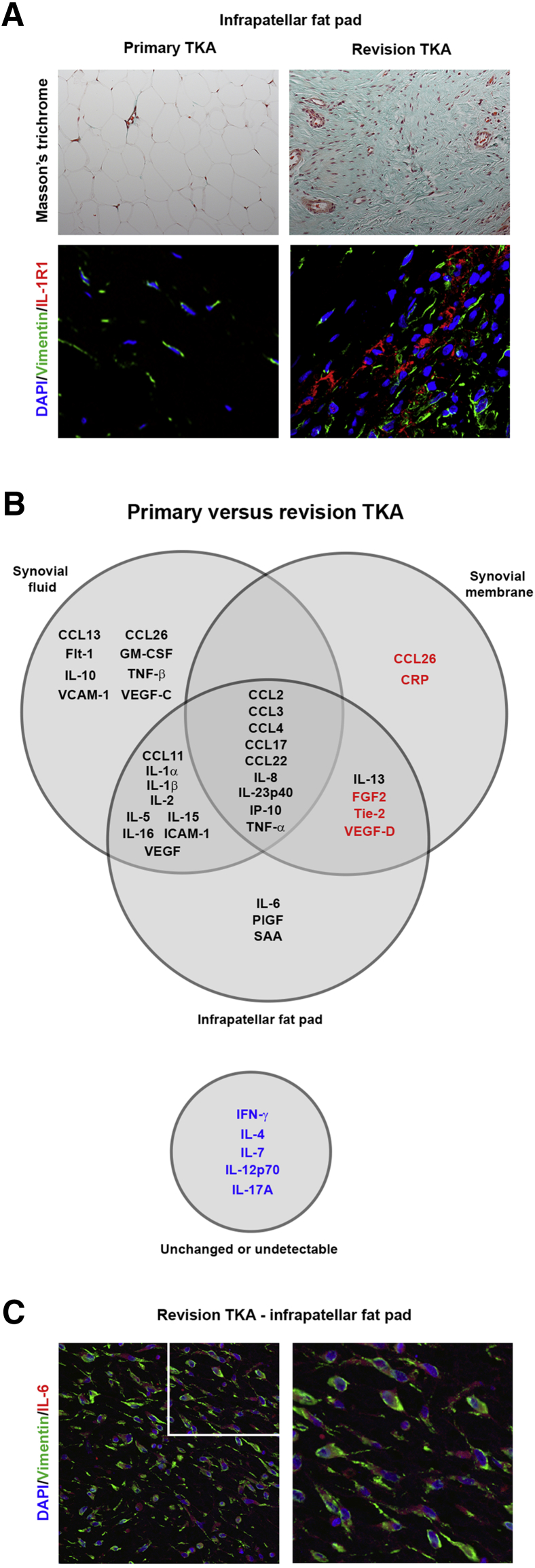
Synovial fluid and tissues isolated from patients undergoing revision total knee arthroplasty (TKA) are characterized by dramatic tissue remodeling and chronic inflammation. **A:** Representative images of Masson trichrome and IL-1R1 (green [fluorescein isothiocyanate (FITC)])/vimentin (red [tetrarhodamine isothiocyanate (TRITC)]) stained infrapatellar fat pad from patients undergoing primary TKA and patients undergoing revision TKA. There is a significant increase in collagen and IL-1R1 expression in revision TKA tissue compared with primary TKA tissue, and IL-1R1 is predominantly expressed on elongated bipolar mesenchymal cells in revision TKA tissue. Images acquired on a Nikon inverted microscope and a Nikon A1R point scanning confocal microscope, respectively. **B:** Venn diagram demonstrating change in protein expression in synovial fluid, infrapatellar fat pad, and synovial membrane isolated from patients undergoing revision TKA compared with patients undergoing primary TKA. Protein expression was quantified using human V-Plex electrochemiluminescence detection kits from MesoScaleDiscovery. Markers in black text are significantly elevated in revision TKA, markers in blue text are not significantly different or undetectable, and markers in red text are significantly decreased in revision TKA. Significance was taken as *P* < 0.05. **C:** Representative images of vimentin [green (FITC)]– and IL-6 [red (TRITC)]–stained infrapatellar fat pad from patients undergoing revision TKA. There are a significant number of vimentin and IL-6 dual-positive fibroblasts in post-TKA infrapatellar fat pad. Images acquired on a Nikon A1R point scanning confocal microscope. Original magnification, ×20 (**A** and **C**); ×40 (**inset**). CRP, C-reactive protein; FGF, fibroblast growth factor; Flt, Fms-like tyrosine kinase 1; GM-CSF, granulocyte-macrophage colony-stimulating factor; ICAM, intercellular adhesion molecule; IFN, interferon; IP-10, interferon gamma-induced protein 10; PIGF, placental growth factor; SAA, serum amyloid A; Tie, tyrosine kinase; TNF, tumor necrosis factor; VCAM, vascular cell adhesion molecule; VEGF, vascular endothelial growth factor.

**Figure 2:**
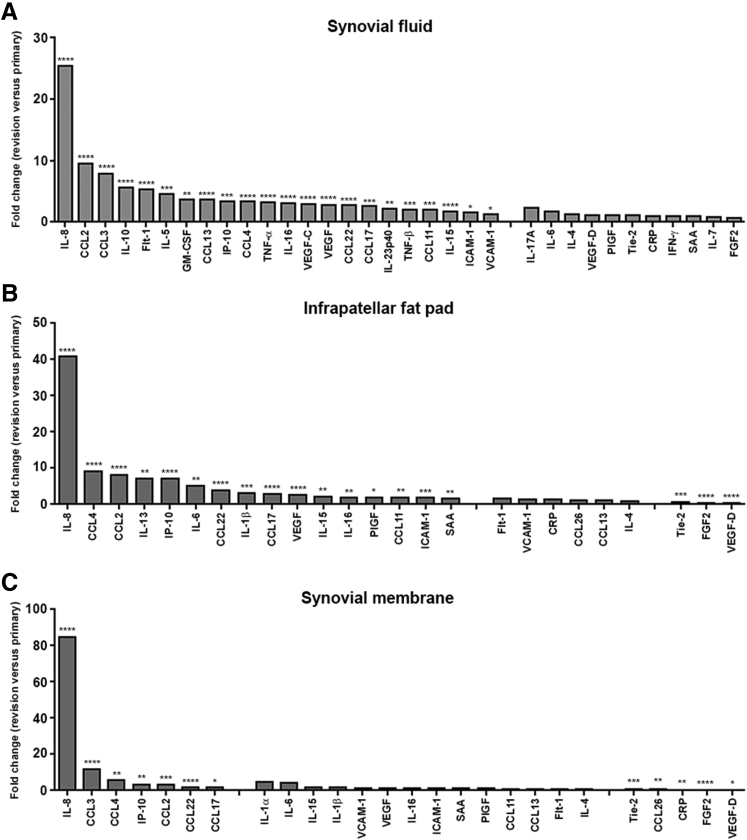
Markers of inflammation are elevated in patients undergoing revision total knee arthroplasty (TKA). Fold change in protein expression in synovial fluid (**A**), infrapatellar fat pad (**B**), and synovial membrane (**C**) isolated from patients undergoing revision TKA compared with patients undergoing primary TKA. Fold change was calculated using median protein expression. Significance was calculated using *U*-test. ^∗^*P* < 0.05, ^∗∗^*P* < 0.01, ^∗∗∗^*P* < 0.001, and ^∗∗∗∗^*P* < 0.0001 primary versus revision patients. CRP, C-reactive protein; FGF, fibroblast growth factor; Flt, Fms-like tyrosine kinase 1; GM-CSF, granulocyte-macrophage colony-stimulating factor; ICAM, intercellular adhesion molecule; IFN, interferon; IP-10, interferon gamma-induced protein 10; PIGF, placental growth factor; SAA, serum amyloid A; Tie, tyrosine kinase; TNF, tumor necrosis factor; VCAM, vascular cell adhesion molecule; VEGF, vascular endothelial growth factor.

**Supplemental Figure S1:**
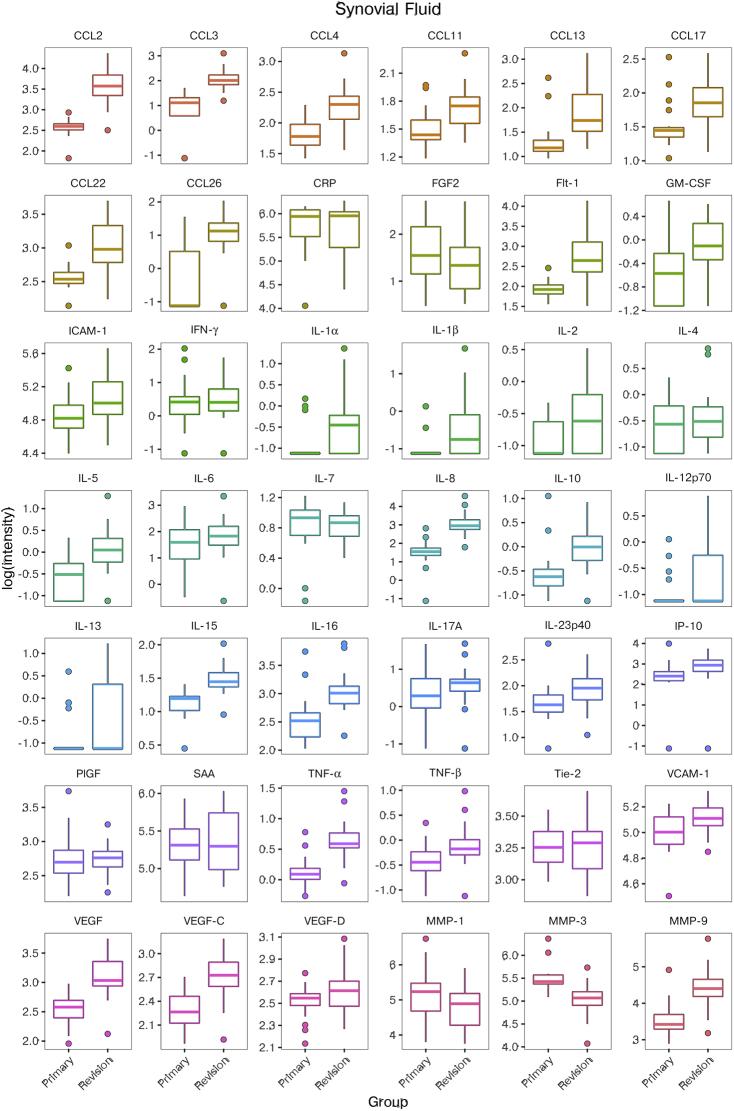
Protein expression profile of synovial fluid isolated from patients undergoing primary and revision total knee arthroplasty. Data are presented as box-and-whiskers plot. CRP, C-reactive protein; FGF, fibroblast growth factor; Flt, Fms-like tyrosine kinase 1; GM-CSF, granulocyte-macrophage colony-stimulating factor; ICAM, intercellular adhesion molecule; IFN, interferon; IP-10, interferon gamma-induced protein 10; MMP, matrix metalloproteinase; PIGF, placental growth factor; SAA, serum amyloid A; Tie, tyrosine kinase; TNF, tumor necrosis factor; VCAM, vascular cell adhesion molecule; VEGF, vascular endothelial growth factor.

**Supplemental Figure S2:**
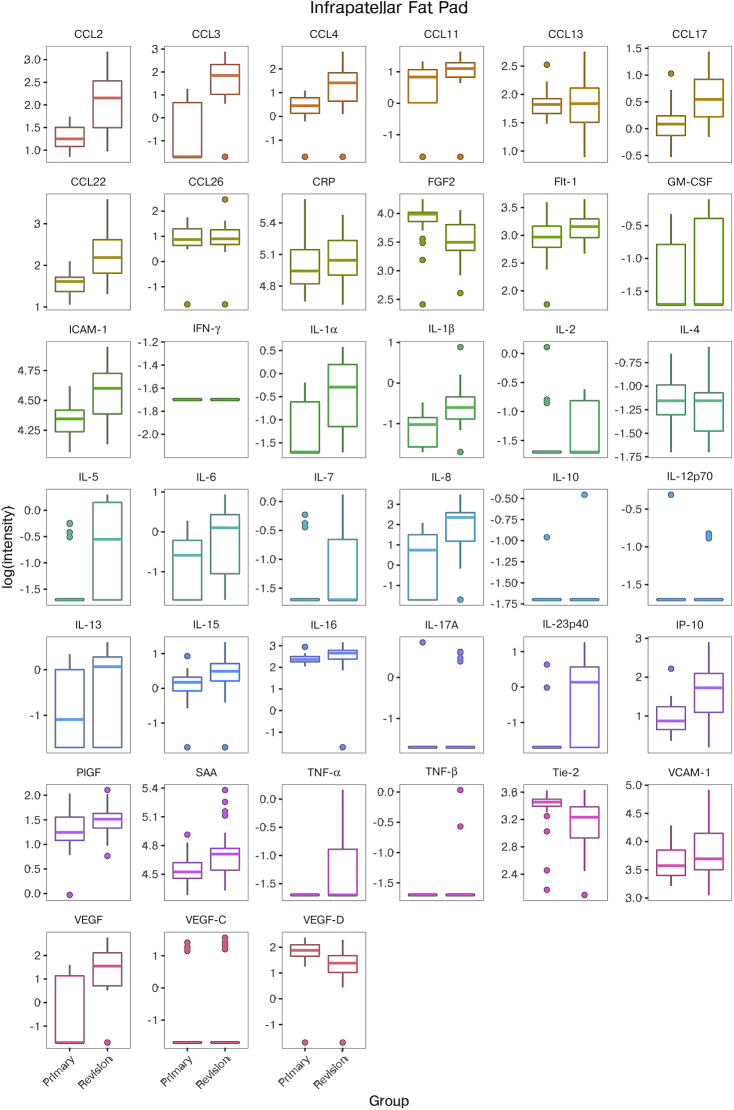
Protein expression profile of infrapatellar fat pad isolated from patients undergoing primary and revision total knee arthroplasty. Data are presented as box-and-whiskers plot. CRP, C-reactive protein; FGF, fibroblast growth factor; Flt, Fms-like tyrosine kinase 1; GM-CSF, granulocyte-macrophage colony-stimulating factor; ICAM, intercellular adhesion molecule; IFN, interferon; IP-10, interferon gamma-induced protein 10; PIGF, placental growth factor; SAA, serum amyloid A; Tie, tyrosine kinase; TNF, tumor necrosis factor; VCAM, vascular cell adhesion molecule; VEGF, vascular endothelial growth factor.

**Supplemental Figure S3:**
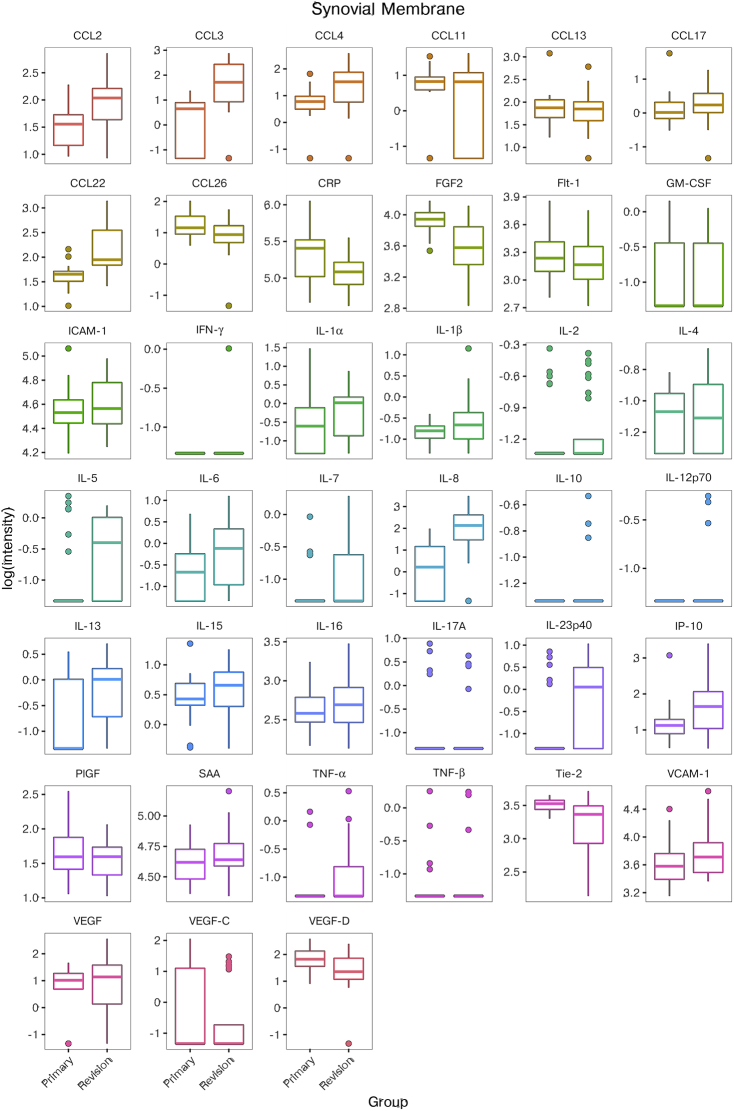
Protein expression profile of synovial membrane isolated from patients undergoing primary and revision total knee arthroplasty. Data are presented as box-and-whiskers plot. CRP, C-reactive protein; FGF, fibroblast growth factor; Flt, Fms-like tyrosine kinase 1; GM-CSF, granulocyte-macrophage colony-stimulating factor; ICAM, intercellular adhesion molecule; IFN, interferon; IP-10, interferon gamma-induced protein 10; PIGF, placental growth factor; SAA, serum amyloid A; Tie, tyrosine kinase; TNF, tumor necrosis factor; VCAM, vascular cell adhesion molecule; VEGF, vascular endothelial growth factor.

The post-TKA joint is associated with the persistence of fibroblasts and extensive fibrotic remodeling of the replacement joint (Figure 1A).^22^, ^35^ To determine the degree to which TKA is associated with inflammation, synovial fluid, synovial membrane, and infrapatellar fat pad were collected from patients undergoing primary or revision surgery and the expression of 39 soluble inflammatory proteins was profiled at each anatomical location. When comparing protein expression between pre- and post-TKA samples, it was discovered that all three anatomical locations in the fibrotic joint display an enhanced proinflammatory state (Tables 2, 3, and 4, Figure 2, and Supplemental Figures S1, S2, and S3). Synovial fluid was the most dramatically affected, with elevated expression of 26 inflammatory markers, followed by the infrapatellar fat pad (22 up-regulated proteins) and the synovial membrane (10 up-regulated proteins). These data were used to construct a Venn diagram (Figure 1B) illustrating the anatomical location of the up-regulated inflammatory markers. CCL2, CCL3 (MIP-1α), CCL4 (MIP-1β), CCL17 (TARC), CCL22 (MDC), IL-8, IP-10, IL-23p40, and tumor necrosis factor-α were up-regulated in all three regions of the fibrotic joint. By far the most induced protein was IL-8, which was expressed at approximately 35-fold, approximately 40-fold, and approximately 80-fold higher levels in the synovial fluid, infrapatellar fat pad, and synovial membrane, respectively (Figure 2). The synovial fluid was the most proinflammatory, with eight proteins exclusively elevated in revision TKA compared with primary TKA. These proteins were the granulocyte recruitment ligands CCL13 (monocyte chemoattractant protein-4) and CCL26 (eotaxin-3), the granulocyte modulator granulocyte-macrophage colony-stimulating factor, tumor necrosis factor-β, the cleaved form of vascular cell adhesion molecule 1, and proangiogenic factors vascular endothelial growth factor-C and Fms-like tyrosine kinase 1 (Figure 1B). Together, these markers suggest the synovial fluid of the fibrotic joint resembled a state of chronic allergic inflammation.^36^ Up-regulated T-cell stimulators IL-2, IL-15, and IL-16, the inflammatory cytokines IL-1α, IL-1β, and IL-5, and the cleaved form of intercellular adhesion molecule 1 were common to the synovial fluid and infrapatellar fat pad of the fibrotic joint.

**Table 2:** Synovial Fluid: Primary TKA versus Revision TKA

Marker	Primary TKA		Revision TKA		Revision versus Primary TKA	
	Median, pg/mL	Range, pg/mL	Median, pg/mL	Range, pg/mL	Fold change	*P* value
Significantly increased						
IL-8	35	0–655	898	62–36,728	25.49	<0.0001
CCL2	395	67–857	3744	318–23,297	9.48	<0.0001
CCL3	13	0–51	102	15–1257	7.93	<0.0001
IL-10	0.16	0–11.2	0.92	0–8.3	5.58	<0.0001
Flt-1	84	36–288	448	33–13,621	5.35	<0.0001
IL-5	0.23	0–2.1	1.05	0–19.4	4.56	<0.001
GM-CSF	0.19	0–4.5	0.71	0–4.0	3.71	<0.01
CCL13	15.0	9–415	54.8	14–1320	3.67	<0.0001
IP-10	256	130–9980	873	196–5562	3.41	<0.001
CCL4	60	27–195	200	36–1358	3.33	<0.0001
TNF-α	1.16	0.5–5.9	3.81	0.8–28.1	3.30	<0.0001
IL-16	331	107–5551	1022	180–7667	3.09	<0.0001
VEGF-C	184	74–509	534	83–1542	2.90	<0.0001
VEGF	379	91–948	1080	133–5555	2.85	<0.0001
CCL22	343	138–1087	954	174–4997	2.78	<0.0001
CCL17	28.0	11–337	71.7	14–384	2.56	<0.001
IL-23p40	42.8	6–652	90.2	11–403	2.11	<0.01
TNF-β	0.28	0–2.1	0.59	0–9.5	2.08	<0.001
CCL11	27.3	15–94	56.0	23–206	2.05	<0.001
IL-15	15.6	3–25	27.9	9–104	1.79	<0.0001
ICAM-1	66,071	25,024–265,365	101,434	31,383–458,122	1.54	<0.05
VCAM-1	100,705	31,963–167,370	128,900	70,772–210,136	1.28	<0.05
CCL26	0.0	0–36	13.3	0–107	NA	<0.0001
IL-1β	0.00	0–1.3	0.10	0–47.1	NA	<0.001
IL-1α	0.00	0–1.4	0.28	0–22.9	NA	<0.01
IL-2	0.00	0–0.4	0.17	0–3.2	NA	<0.05
Not significantly different						
IL-17	1.87	0–47	4.28	1–49	2.29	0.23
IL-6	39.1	0–921	67.3	0–2236	1.72	0.18
IL-4	0.20	0–2.0	0.24	0–7.6	1.20	0.64
VEGF-D	352	137–593	411	185–1215	1.17	0.16
PlGF	497	159–5463	576	179–1777	1.16	0.67
Tie-2	1797	967–3547	1951	745–4970	1.09	0.95
CRP	875,587	11,385–1,421,021	902,869	25,449–1,865,959	1.03	0.72
IFN-γ	2.54	0–105	2.53	0–55	1.00	0.88
SAA	205,119	43,141–851,868	198,230	57,469–1,072,174	0.97	0.81
IL-7	8.49	0.7–16.5	7.30	2.5–13.6	0.86	0.29
FGF2	35.2	3–512	21.6	3–495	0.61	0.25
IL-12p70	0.00	0–1.1	0.00	0–7.5	NA	0.11
IL-13	0.00	0–3.9	0.00	0–16.5	NA	0.09

CRP, C-reactive protein; FGF, fibroblast growth factor; Flt, Fms-like tyrosine kinase 1; GM-CSF, granulocyte-macrophage colony-stimulating factor; ICAM, intercellular adhesion molecule; IFN, interferon; IP-10, interferon gamma-induced protein 10; NA, not applicable; PIGF, placental growth factor; SAA, serum amyloid A; Tie, tyrosine kinase; TKA, total knee arthroplasty; TNF, tumor necrosis factor; VCAM, vascular cell adhesion molecule; VEGF, vascular endothelial growth factor.

**Table 3:** Infrapatellar Fat Pad: Primary TKA versus Revision TKA

Marker	Primary TKA		Revision TKA		Revision versus Primary TKA	
	Median, pg/mL	Range, pg/mL	Median, pg/mL	Range, pg/mL	Fold change	*P* value
Significantly increased						
IL-8	6	0–117	225	0–2952	40.80	<0.0001
CCL4	2.8	0–12	26.0	0–522	9.25	<0.0001
CCL2	18	7–54	142	9–1491	8.03	<0.0001
IL-13	0.16	0–2.2	1.15	0–4.0	7.18	<0.01
IP-10	7.4	2–165	53.2	2–786	7.16	<0.0001
IL-6	0.24	0–1.9	1.25	0–8.5	5.15	<0.01
CCL22	41	12–125	154	21–3827	3.76	<0.0001
IL-1β	0.07	0–0.3	0.23	0–7.6	3.19	<0.001
CCL17	1.20	0–11	3.51	1–27	2.91	<0.0001
VEGF	22.5	0–83	61.5	8–1616	2.74	<0.0001
IL-15	1.47	0–8	3.09	0–21	2.10	<0.01
IL-16	233	112–890	459	0–1430	1.97	<0.01
PlGF	17.6	1–107	32.7	6–128	1.86	<0.05
CCL11	6.8	0–28	12.6	0–43	1.85	<0.01
ICAM-1	22,148	11,699–41,389	39,918	13,665–88,209	1.80	<0.001
SAA	33,446	19,242–82,008	51,401	21,488–239,797	1.54	<0.01
IL-23p40	0	0–4.3	1.35	0–18.2	NA	<0.0001
IL-1α	0	0–0.6	0.49	0–3.7	NA	<0.0001
IL-2	0	0–1.3	0.00	0–0.2	NA	<0.05
IL-5	0	0–0.6	0.26	0–2.0	NA	<0.001
CCL3	0	0–18	70.3	0–755	NA	<0.0001
TNF-α	0	0–0	0.00	0–1.4	NA	<0.01
Not significantly different						
Flt-1	932	57–3910	1436	471–4431	1.54	0.06
VCAM-1	3748	1647–19,192	4964	1134–82,268	1.32	0.09
CRP	87,863	45,144–419,932	111,126	42,185–298,708	1.26	0.33
CCL26	7.46	0–55	7.92	0–288	1.06	0.90
CCL13	66.5	31–335	68.9	8–565	1.04	0.94
IL-4	0.05	0–0.2	0.05	0–0.2	0.95	0.28
GM-CSF	0	0–0.4	0	0–0.8	NA	0.09
IFN-γ	0	0–0	0	0–0	NA	>0.99
IL-10	0	0–0.1	0	0–0.3	NA	>0.99
IL-12p70	0	0–0.5	0	0–0.1	NA	0.62
IL-17A	0	0–7.1	0	0–4.2	NA	0.36
IL-7	0	0–0.6	0	0–1.3	NA	0.09
TNF-β	0	0–0	0	0–1.1	NA	0.49
VEGF-C	0	0–26	0	0–36.6	NA	0.51
Significantly reduced						
Tie-2	2849	149–4208	1712	125–4280	0.60	<0.001
FGF2	9734	258–17,546	3141	408–11,371	0.32	<0.0001
VEGF-D	76.7	0–237	24.3	0–193	0.32	<0.0001

CRP, C-reactive protein; FGF, fibroblast growth factor; Flt, Fms-like tyrosine kinase 1; GM-CSF, granulocyte-macrophage colony-stimulating factor; ICAM, intercellular adhesion molecule; IFN, interferon; IP-10, interferon gamma-induced protein 10; NA, not applicable; PIGF, placental growth factor; SAA, serum amyloid A; Tie, tyrosine kinase; TKA, total knee arthroplasty; TNF, tumor necrosis factor; VCAM, vascular cell adhesion molecule; VEGF, vascular endothelial growth factor.

**Table 4:** Synovial Membrane: Primary TKA versus Revision TKA

Marker	Primary TKA		Revision TKA		Revision versus Primary TKA	
	Median, pg/mL	Range, pg/mL	Median, pg/mL	Range, pg/mL	Fold change	*P* value
Significantly increased						
IL-8	2	0–97	135	0–3042	84.50	<0.0001
CCL3	4.4	0–24	51.7	0–750	11.74	<0.0001
CCL4	6.0	0–65	33.0	0–380	5.53	<0.01
IP-10	13.3	3–1183	44.7	3–2507	3.37	<0.01
CCL2	36	9–191	109	8–720	3.03	<0.001
CCL22	45.0	10–145	88.7	26–1389	1.97	<0.0001
CCL17	0.99	0–58	1.68	0–19	1.69	<0.05
IL-23p40	0	0–7.1	1.08	0–10.8	NA	<0.001
IL-13	0	0–3.5	0.98	0–5.1	NA	<0.01
TNF-α	0	0–1.4	0.00	0–3.3	NA	<0.05
Not significantly different						
IL-1α	0.20	0–30	1.00	0–7	4.98	0.08
IL-6	0.17	0–4.8	0.72	0–12.6	4.28	0.05
IL-15	2.65	0–23)	4.51	0–18	1.71	0.25
IL-1β	0.11	0–0.3	0.17	0–14.1	1.54	0.09
VCAM-1	3799	1420–25,228	5163	2311–45,561	1.36	0.09
VEGF	10.3	0–46	13.8	0–362	1.33	0.94
IL-16	382	147–1743	493	135–3003	1.29	0.21
ICAM-1	33,978	15,606–115,625	36,721	17,683–95,444	1.08	0.25
SAA	41,586	22,888–84,910	43,694	21,989–159,994	1.05	0.21
PlGF	39.4	11–353	39.8	11–116	1.01	0.81
CCL11	6.61	0–34	6.53	0–41	0.99	0.36
CCL13	75.0	17–1199	70.7	6–609	0.94	0.80
Flt-1	1727	646–7201	1466	526–5691	0.85	0.32
IL-4	0.04	0–0.1	0.03	0–0.2	0.81	0.90
GM-CSF	0	0–1.4	0	0–1.1	NA	>0.99
IFN-γ	0	0–0.2	0	0–1.0	NA	>0.99
IL-10	0	0–0	0	0–0.2	NA	0.24
IL-12p70	0	0–0.1	0	0–0.5	NA	0.24
IL-17A	0	0–7.7	0	0–4.3	NA	0.81
IL-2	0	0–0.4	0	0–0.4	NA	0.45
IL-5	0	0–2.2	0.36	0–1.5	NA	0.07
IL-7	0	0–0.9	0	0–1.9	NA	0.09
TNF-β	0	0–1.7	0	0–1.7	NA	0.64
VEGF-C	0	0–114	0	0–30	NA	0.60
Significantly reduced						
Tie-2	3364	2006–4518	2322	138–5181	0.69	<0.001
CCL26	14.4	4–104	8.7	0–55	0.61	<0.01
CRP	255,593	46,461–1,128,993	121,677	42,048–355,751	0.48	<0.01
FGF2	8779	3453–15,081	3781	679–13,018	0.43	<0.0001
VEGF-D	66.5	8–390	22.7	0–249	0.34	<0.05

CRP, C-reactive protein; FGF, fibroblast growth factor; Flt, Fms-like tyrosine kinase 1; GM-CSF, granulocyte-macrophage colony-stimulating factor; ICAM, intercellular adhesion molecule; IFN, interferon; IP-10, interferon gamma-induced protein 10; NA, not applicable; PIGF, placental growth factor; SAA, serum amyloid A; Tie, tyrosine kinase; TKA, total knee arthroplasty; TNF, tumor necrosis factor; VCAM, vascular cell adhesion molecule; VEGF, vascular endothelial growth factor.

### Fibroblasts from the Infrapatellar Fat Pad Polarize to a Proinflammatory Phenotype in Response to IL-1α

**Figure 3:**
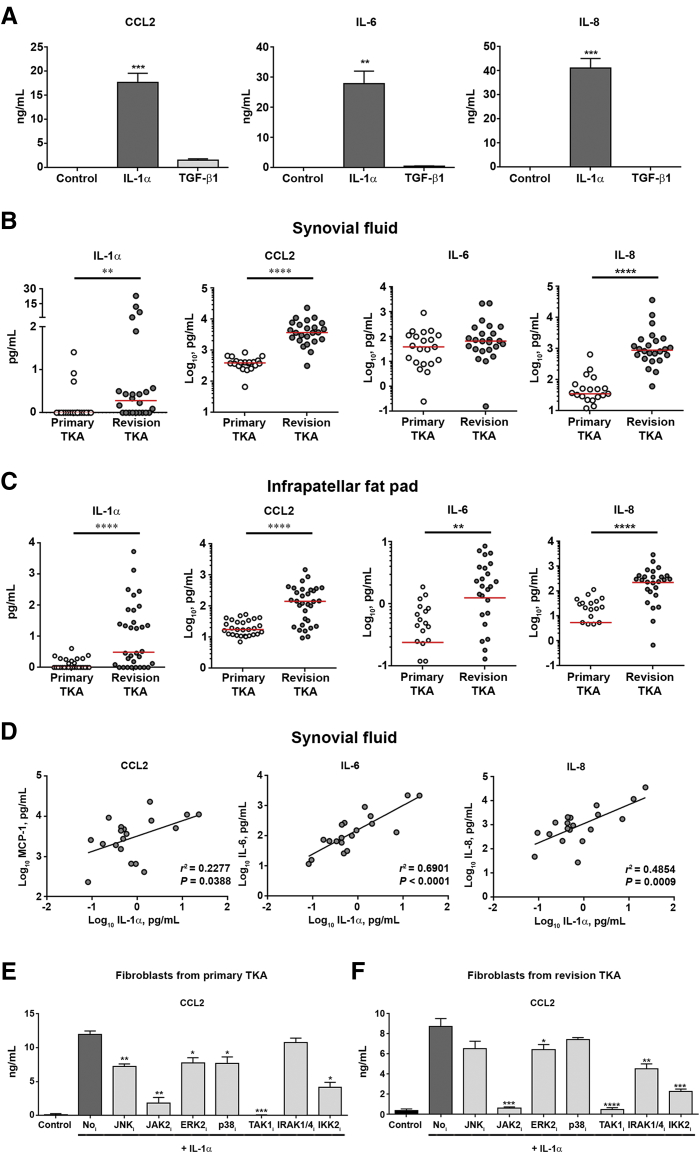
Markers elevated after total knee arthroplasty (TKA) are associated with fibroblast activation. **A:** Fibroblasts isolated from the infrapatellar fat pad of patients undergoing primary TKA were treated with control media, IL-1α (500 pg/mL), or transforming growth factor (TGF)-β1 (3 ng/mL) for 24 hours and secreted levels of CCL2, IL-6, and IL-8, determined by enzyme-linked immunosorbent assay (ELISA). Data are analyzed using paired *t*-tests. **B** and **C:** IL-1α, CCL2, IL-6, and IL-8 protein levels were quantified in synovial fluid (**B**) and infrapatellar fat pad (**C**) and compared between patients undergoing primary and revision TKA. Data are presented as individual patient values, with median signified by the **red line** and analyzed using a *U*-test. **D:** IL-1α protein expression correlates against CCL2, IL-6, and IL-8 levels in synovial fluid. Data are presented as individual patient values with associated linear regression analysis. **E** and **F:** Fibroblasts isolated from the infrapatellar fat pad of patients undergoing primary (**E**) and revision (**F**) TKA were pretreated for 1 hour with several selective inhibitors [TAK1 inhibitor (TAK_i_), 1 μmol/L; all other inhibitors, 10 μmol/L] before stimulation with IL-1α (500 pg/mL) for 24 hours, and CCL2 was quantified by ELISA. Data are analyzed using paired *t*-tests. Data are presented as means ± SEM (**A**, **E**, and **F**). *n* = 6 (**A**, **E**, and **F**). ^∗^*P* < 0.05, ^∗∗^*P* < 0.01, ^∗∗∗^*P* < 0.001, and ^∗∗∗∗^*P* < 0.0001 primary versus revision patients. ERK, extracellular signal–regulated kinase; IKK, Iκ B kinase; IRAK, IL-1 receptor associated kinase; JAK, Janus activating kinase; JNK, c-Jun N-terminal kinase; MCP, monocyte chemoattractant protein.

Having established that the fibrotic post-TKA joint acquires a state of unresolved inflammation, it was next determined if fibroblasts contribute to this state. We previously reported that the infrapatellar fat pad is a site at which extensive fibrotic remodeling and fibroblast accumulation occur after TKA.^22^ Furthermore, we now demonstrate that fibroblasts in the post-TKA joint express IL-1R1 (Figure 1A). IL-1α polarizes fibroblasts toward a proinflammatory state^19^ and is elevated in both the post-TKA infrapatellar fat pad and synovial fluid (Figure 1B). Fibroblasts isolated from the infrapatellar fat pad and exposed to pg/mL levels of IL-1α underwent a dramatic induction of CCL2, IL-6, and IL-8, all being secreted at ng/mL levels. By contrast, profibrogenic TGF-β1 failed to induce this proinflammatory state (Figure 3A). IL-6 was remarkable in that it was a unique inflammatory feature of the infrapatellar fat pad in the post-TKA joint (Figure 1B). Multiarray protein measurements confirmed that IL-6 is up-regulated in the remodeled infrapatellar fat pad, but remains unchanged in the synovial fluid (Figure 3, B and C). Moreover, elevated IL-1α in the fibrotic infrapatellar fat pad (pg/mL levels) correlated with enhanced expression of CCL2, IL-6, and IL-8 (Figure 3D), suggesting that fibroblasts exposed to IL-1α in the microenvironment of the fibrotic infrapatellar fat pad are likely to be a major source of proinflammatory CCL2, IL-6, and IL-8. In agreement with this, immunocytochemistry performed on the post-TKA infrapatellar fat pad identified a significant number of the vimentin-positive fibroblasts that were also positive for IL-6 (Figure 1C).

**Supplemental Figure S4:**
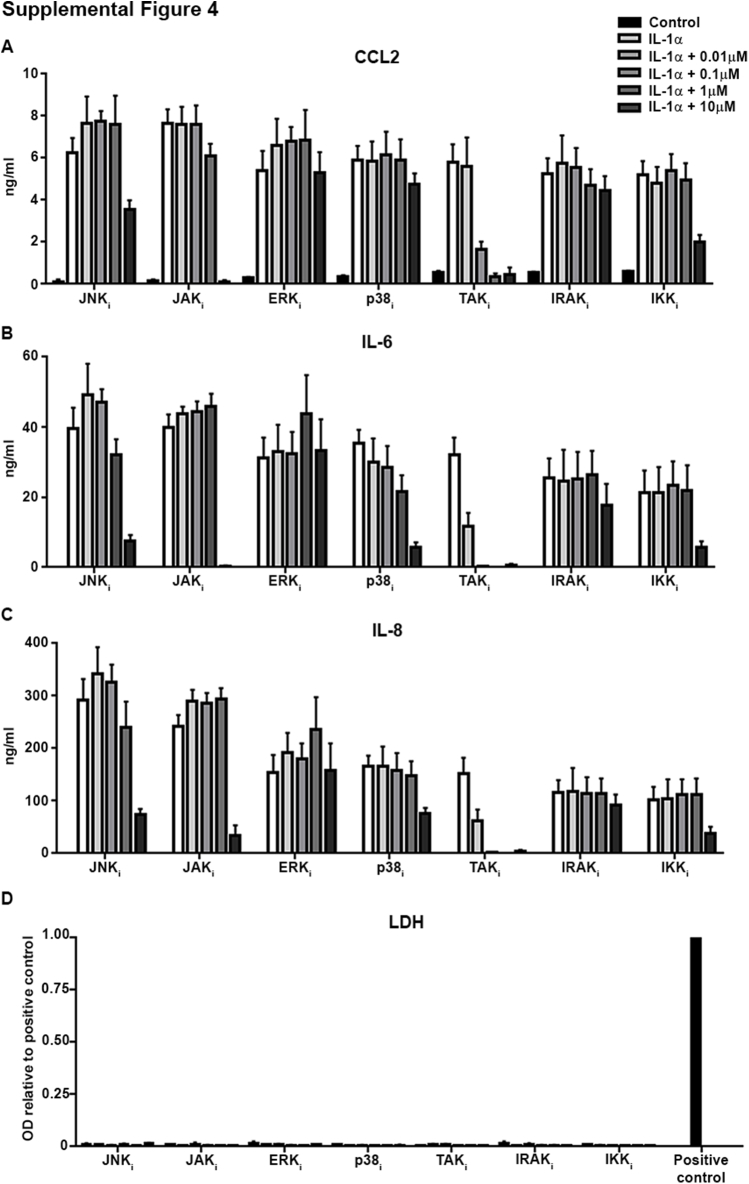
Targeting key intracellular signaling pathways in fibroblasts blocks cytokine secretion. Fibroblasts isolated from the infrapatellar fat pad of patients undergoing primary total knee arthroplasty were pretreated for 1 hour with increasing doses (0.01 to 10 μmol/L) of several selective inhibitors before stimulation with 500 pg/mL IL-1α for 24 hours. The secretion of CCL2 (**A**), IL-6 (**B**), and IL-8 (**C**) was quantified by enzyme-linked immunosorbent assay, and the release of lactate dehydrogenase (**D**) was quantified using a Pierce LDH Cytotoxicity Assay Kit. Data are presented as means ± SEM. *n* = 6 (**A**–**D**). ERK, extracellular signal–regulated kinase; IKK, Iκ B kinase; IRAK, IL-1 receptor associated kinase; JAK, Janus activating kinase; JNK, c-Jun N-terminal kinase; TAK, transforming growth factor β–activated kinase.

To determine intracellular signaling pathways responsible for mediating IL-1α–induced polarization of fibroblasts, the cells were treated with inhibitors of downstream signal transduction molecules. Inhibitors of Janus activating kinase 2, transforming growth factor β–activated kinase 1 (TAK1), and IKK-2, signaling molecules known to activate the cardinal proinflammatory transcription factor NF-κB,^37^, ^38^, ^39^ blunted IL-1α–induced expression of CCL2 by twofold or greater (Figure 3, E and F, and Supplemental Figure S4).

**Figure 4:**
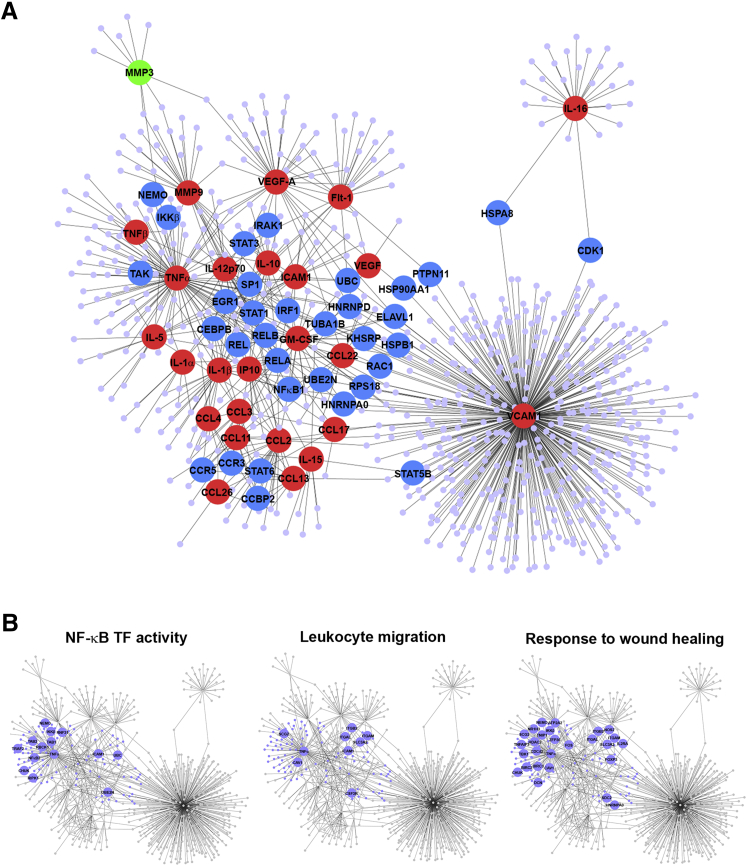
Network and module analysis. **A:** Protein-protein interaction network built from differentially expressed markers. Red indicates up-regulated marker protein; green, down-regulated marker protein; blue, proteins with either degree (number of interactions) ≥5, betweenness centrality ≥0.01, or one of a selected set of proteins of interest [transforming growth factor β–activated kinase, IL-1 receptor associated kinase (IRAK) 1, STAT3, Iκ B kinase (IKK) β, or NF-kappa-B essential modulator]. **B:** Network with one module (highly connected subnetwork) highlighted in purple. Large labeled nodes show proteins associated with selected enriched gene ontology biological processes mapped to the module; highlighted nodes are associated with the processes positive regulation of NF-κB transcription factor activity, leukocyte migration, and response to wound healing. CCBP, chemokine-binding protein 2; CEBPB, CCAAT/enhancer binding protein beta; EGR, early growth response protein 1; GM-CSF, granulocyte-macrophage colony-stimulating factor; HSP, heat shock protein; MMP, matrix metalloproteinase; TNF, tumor necrosis factor; VEGF, vascular endothelial growth factor.

To investigate the *in vivo* relevance of these data, a systems bioinformatics analysis was performed using the protein expression data derived from the multiarray protein measurements of up-regulated proinflammatory molecules in synovial fluid of the post-TKA joint (Table 2 and Figure 1B). By using a network reconstruction technique, the innate immune protein interactome of the remodeled TKA joint was interrogated (Figure 4). Of particular note was the mapping of NF-κB family proteins Rel (c-Rel), RelA (p65), RelB, and NF-κB1 (p50) in a network including CCL17, IP-10, CCL2, CCL22, CCL3, CCL4, CCL11 (eotaxin), and IL-15, all of which were identified as up-regulated in one or both of the synovial fluid and infrapatellar fat pad of the fibrotic post-TKA joint (Figure 4A). The reconstructed network also contained TAK1, IKK-2, and the Janus activating kinase 2 target STAT3, rationalizing their role in the CCL2 response to IL-1α. Proteins in the network and in highly connected submodules were also enriched for gene ontology processes, including leukocyte migration and response to wound healing (Figure 4B).

### Inflammatory Fibroblasts Regulate Monocyte Recruitment via NF-κB–Regulated Expression of CCL2

**Figure 5:**
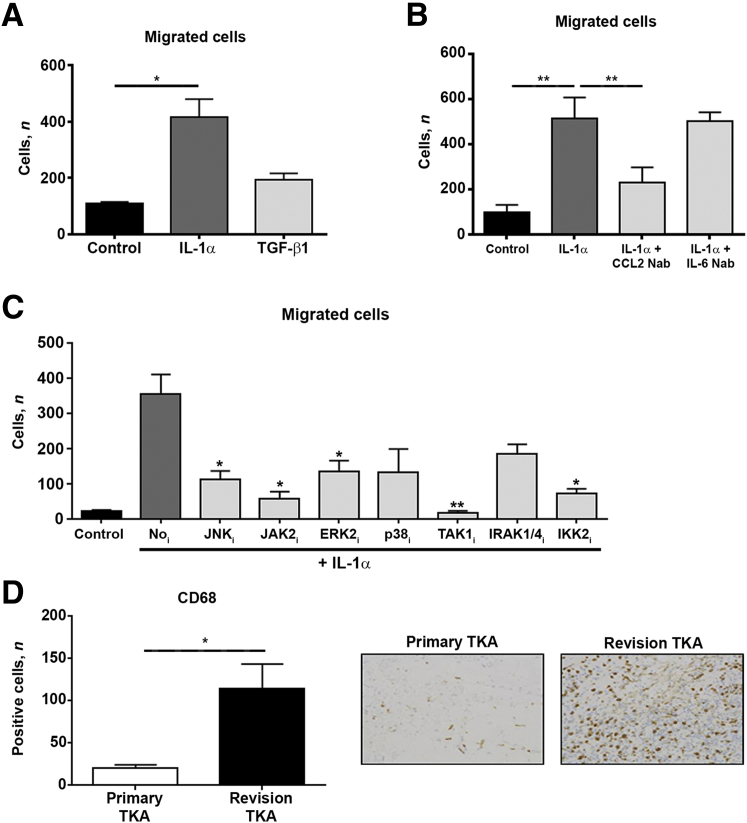
IL-1α–activated fibroblasts promote recruitment of monocytes by secreting CCL2. Fibroblasts isolated from the infrapatellar fat pad of patients undergoing primary total knee arthroplasty (TKA) were stimulated with control media, IL-1α (500 pg/mL), or transforming growth factor (TGF)-β1 (3 ng/mL) for 24 hours to generate conditioned media for transwell migration assays. **A:** Number of monocytes recruited by conditioned media from unstimulated (control), IL-1α–stimulated, and TGF-β1–stimulated fibroblasts. **B:** Conditioned medium from IL-1α–stimulated fibroblasts was pretreated for 1 hour with neutralizing antibodies against CCL2 and IL-6 and then used in transwell migration assays. **C:** Conditioned medium from fibroblasts pretreated for 1 hour with several selective inhibitors (transforming growth factor β–activated kinase, 1 μmol/L; all other inhibitors, 10 μmol/L) before stimulation with IL-1α for 24 hours and then used in transwell migration assays. The number of migrated cells was quantified on a Nikon Eclipse microscope. Data are analyzed using paired *t*-tests. **D:** Number of CD68^+^ cells in the infrapatellar fat pad of patients undergoing primary and revision TKA was quantified in 10× randomly selected high-powered fields using Nikon NIS elements image analysis software. Data are analyzed using *U*-test. Data are presented as means ± SEM (**C** and **D**). *n* = 6 (**A**–**C**). ^∗^*P* < 0.05, ^∗∗^*P* < 0.01 versus No_i_. Original magnification, ×20 (**D**). ERK, extracellular signal–regulated kinase; IKK, Iκ B kinase; IRAK, IL-1 receptor associated kinase; JAK, Janus activating kinase; JNK, c-Jun N-terminal kinase.

Having established that IL-1α polarizes fibroblasts toward an inflammatory phenotype, it was next tested if these cells are able to directly stimulate recruitment of immune cells, specifically monocytes. To test this hypothesis, a transwell migration assay was performed with human THP-1 monocytes and conditioned media from unstimulated, IL-1α–stimulated, or TGF-β1–stimulated fibroblasts. Conditioned media from IL-1α–stimulated fibroblasts significantly increased migration of monocytes compared with unstimulated cells. TGF-β1 treatment had no effect on monocyte migration (Figure 5A). Pretreating the conditioned media with neutralizing antibodies against CCL2 and IL-6 identified CCL2 as the predominant mediator of monocyte recruitment, such that anti-CCL2 reduced monocyte recruitment by approximately 80%; in contrast, anti–IL-6 had negligible effects (Figure 5B). Treatment of IL-1α–stimulated fibroblasts with inhibitors of Janus activating kinase 2, TAK1, and IKK effectively prevented their ability to recruit monocytes, confirming the requirement of NF-κB activation (Figure 5C). Because CCL2 was found to be significantly elevated in the post-TKA synovial fluid and infrapatellar fat pad and is integrated with NF-κB in the innate immune interactome of the fibrotic joint, CD68^+^ monocytes in the fibrotic joint were quantified. There was a significant increase in the number of CD68^+^ monocytes in the infrapatellar fat pad of fibrotic revision post-TKA joints compared with nonfibrotic primary TKA joints (Figure 5D).

### Elevated CCL2 in the Inflammatory Environment of the Fibrotic Post-TKA Joint Correlates with Pain

**Figure 6:**
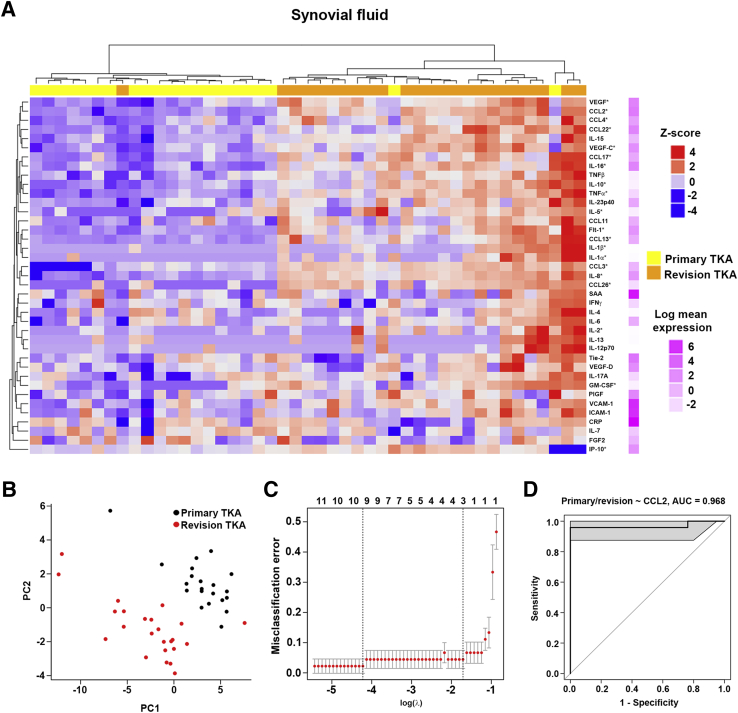
CCL2 in synovial fluid can be used to stratify patients undergoing revision total knee arthroplasty (TKA) from patients undergoing primary TKA. **A:** Heat map of log-transformed protein intensity in synovial fluid isolated from patients undergoing primary or revision TKA clustered using the differentially expressed markers with the highest fold change. **Asterisks** indicate markers that were used for clustering analysis/stratification. **B:** Principal component analysis plot of all patients using all markers. **C:** Classification error as function of the number of markers included, cross validated using glmnet package. **Dotted lines** mark two important values of the regularization parameter λ, which controls the number of markers included. On the left is the value of λ that gives the minimum mean cross-validated error. On the right is the value that gives the most regularized model, such that error is within one SEM of the minimum. **D:** Receiver operating characteristic curve for classification with CCL2 alone [area under the curve (AUC), 0.968; 95% CI, 0.89–1.0]. CRP, C-reactive protein; FGF, fibroblast growth factor; Flt, Fms-like tyrosine kinase 1; GM-CSF, granulocyte-macrophage colony-stimulating factor; ICAM, intercellular adhesion molecule; IFN, interferon; IP-10, interferon gamma-induced protein 10; PIGF, placental growth factor; SAA, serum amyloid A; Tie, tyrosine kinase; TNF, tumor necrosis factor; VCAM, vascular cell adhesion molecule; VEGF, vascular endothelial growth factor.

**Supplemental Figure S5:**
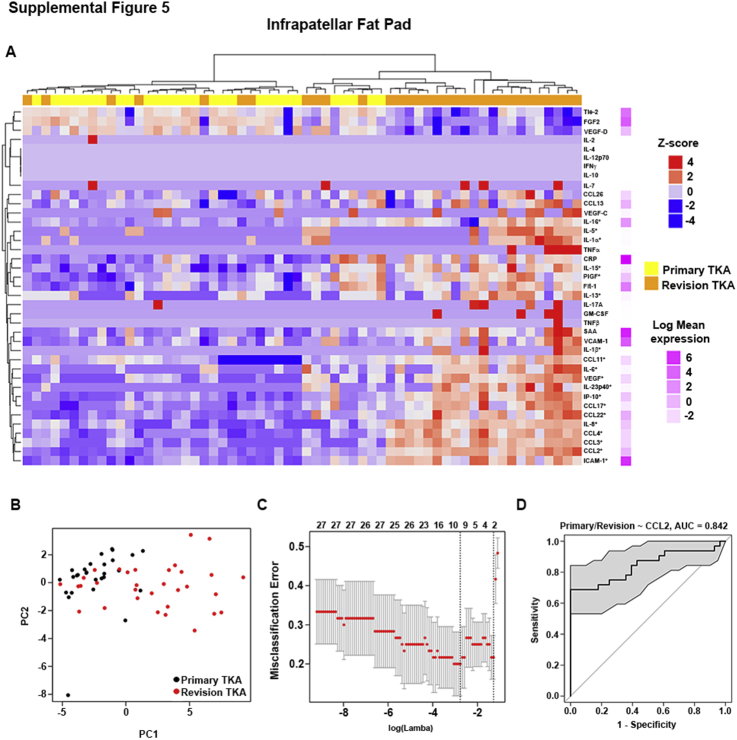
The inflammatory signature in infrapatellar fat pad does not effectively stratify patients into those undergoing revision total knee arthroplasty (TKA) from those undergoing primary TKA. **A:** Heat map of log-transformed protein intensity in infrapatellar fat pad isolated from patients undergoing primary or revision TKA clustered using the DE markers with the highest fold change. **Asterisks** indicate markers that were used for clustering analysis/stratification. **B:** Principal component analysis plot of all patients using all markers. **C:** Classification error as function of the number of markers included, cross validated using glmnet package. **Dotted lines** mark two important values of the regularization parameter λ, which controls the number of markers included. On the left is the value of λ that gives the minimum mean cross-validated error. On the right is the value that gives the most regularized model, such that error is within one SEM of the minimum. **D:** Receiver operating characteristic curve for classification with CCL2 alone [area under the curve (AUC), 0.842; 95% CI, 0.74–0.95]. CRP, C-reactive protein; FGF, fibroblast growth factor; Flt, Fms-like tyrosine kinase 1; GM-CSF, granulocyte-macrophage colony-stimulating factor; ICAM, intercellular adhesion molecule; IFN, interferon; IP-10, interferon gamma-induced protein 10; PIGF, placental growth factor; SAA, serum amyloid A; Tie, tyrosine kinase; TNF, tumor necrosis factor; VCAM, vascular cell adhesion molecule; VEGF, vascular endothelial growth factor.

**Supplemental Figure S6:**
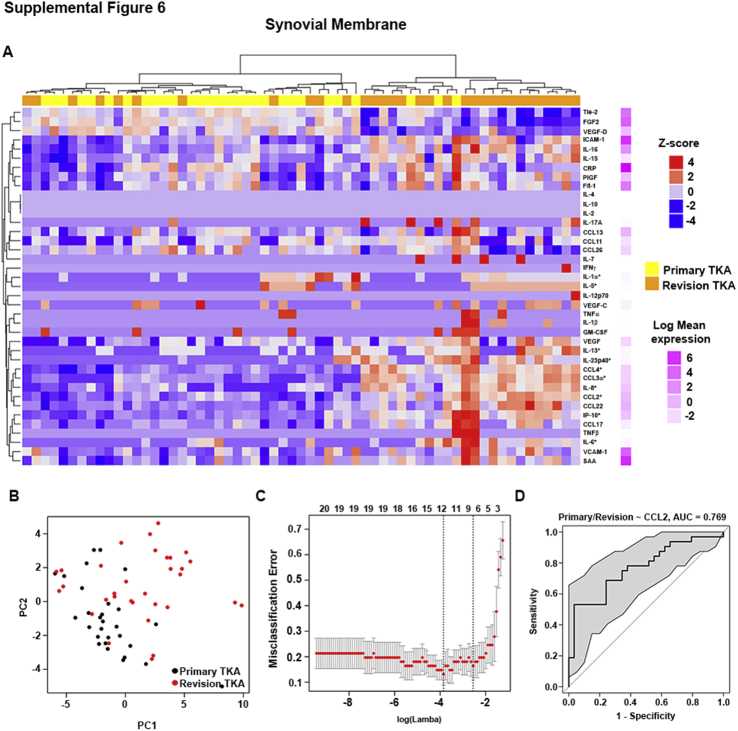
The inflammatory signature in synovial membrane does not effectively stratify patients into those undergoing revision total knee arthroplasty (TKA) from those undergoing primary TKA. **A:** Heat map of log-transformed protein intensity in synovial membrane isolated from patients undergoing primary or revision TKA clustered using the differentially expressed markers with the highest fold change. **Asterisks** indicate markers that were used for clustering analysis/stratification. **B:** Principal component analysis plot of all patients using all markers. **C:** Classification error as function of the number of markers included, cross validated using glmnet package. **Dotted lines** mark two important values of the regularization parameter λ, which controls the number of markers included. On the left is the value of λ that gives the minimum mean cross-validated error. On the right is the value that gives the most regularized model, such that error is within one SEM of the minimum. **D:** Receiver operating characteristic curve for classification with CCL2 alone [area under the curve (AUC), 0.769; 95% CI, 0.65–0.89]. CRP, C-reactive protein; FGF, fibroblast growth factor; Flt, Fms-like tyrosine kinase 1; GM-CSF, granulocyte-macrophage colony-stimulating factor; ICAM, intercellular adhesion molecule; IFN, interferon; IP, interferon gamma-induced protein 10; PIGF, placental growth factor; SAA, serum amyloid A; Tie, tyrosine kinase; TNF, tumor necrosis factor; VCAM, vascular cell adhesion molecule; VEGF, vascular endothelial growth factor.

The degree to which the inflammatory profile of the synovial fluid of the joint stratifies patients into those requiring revision TKA surgery was quantified. Inflammatory proteins with the greatest fold change between primary and revision patients were log transformed and used for hierarchical clustering, which was visualized by a heat map. The use of inflammatory protein expression in the synovial fluid was sufficient to segregate revision from primary TKA patients with only *n* = 1 revision and *n* = 2 primary TKA patients misclassified (Figure 6A). The predictive value of the inflammatory profile of the infrapatellar fat pad (Supplemental Figure S5A) and the synovial membrane (Supplemental Figure S6A) was also assessed. Although a degree of stratification was observed with these tissues, there was a larger degree of overlap between the patient groups compared with synovial fluid. Principal component analysis was performed to further investigate the predictive value of the inflammatory profile in the synovial fluid (Figure 6B). This approach demonstrated the excellent predictive value of the synovial fluid, with distinct separation of primary and revision TKA samples (only *n* = 1 revision TKA patient clustering incorrectly). Similar analysis of the infrapatellar fat pad (Supplemental Figure S5B) and synovial membrane (Supplemental Figure S6B) again demonstrated a much larger degree of overlap compared with synovial fluid. The R package glmnet was used to select a reduced set of inflammatory markers that reliably classify primary and revision TKA patients. Again, the synovial fluid had the most predictive value and indicated that a single inflammatory marker, CCL2, achieved a misclassification error of <5% (Figure 6C). Indeed, using CCL2 alone to generate a receiver operating characteristic curve achieved an area under the curve of 96.8% (95% CI, 89%–100%) in stratifying patients as primary or revision TKA (Figure 6D). The misclassification error was significantly higher for the infrapatellar fat pad (Supplemental Figure S5C) and synovial membrane (Supplemental Figure S6C), whereas the ability of CCL2 alone to stratify patients was also reduced (infrapatellar fat pad: area under the curve, 84.2%; 95% CI, 74%–95%) (Supplemental Figure S5D) (synovial membrane: area under the curve, 76.9%; 95% CI, 65%–89%) (Supplemental Figure S6D). For these tissues, glmnet analysis suggested that more complex models containing additional markers, such as CCL3, CCL13, CCL22, IL-8, and C-reactive protein, may be better able to stratify patients, but would still not match the accuracy of CCL2 in synovial fluid (Supplemental Figures S5C and S6C).

**Figure 7:**
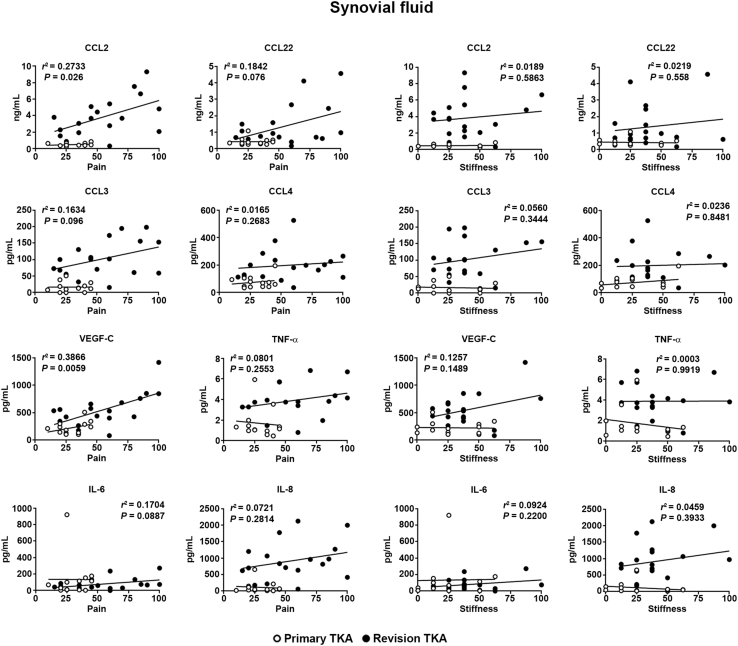
Pain positively correlates with levels of CCL2 in synovial fluid. Pain and stiffness, as determined by Western Ontario and McMaster Universities Osteoarthritis Index scores, were plotted against expression levels of several markers that were significantly elevated in the synovial fluid of patients undergoing revision total knee arthroplasty (TKA). Data are presented for patients undergoing primary and revision TKA, and correlations were determined by linear regression analysis. Increased levels of CCL2 are highly predictive of pain but are poorly correlated to stiffness. *n* = 13 primary TKA group; *n* = 18 revision TKA group. TNF, tumor necrosis factor; VEGF, vascular endothelial growth factor.

**Supplemental Figure S7:**
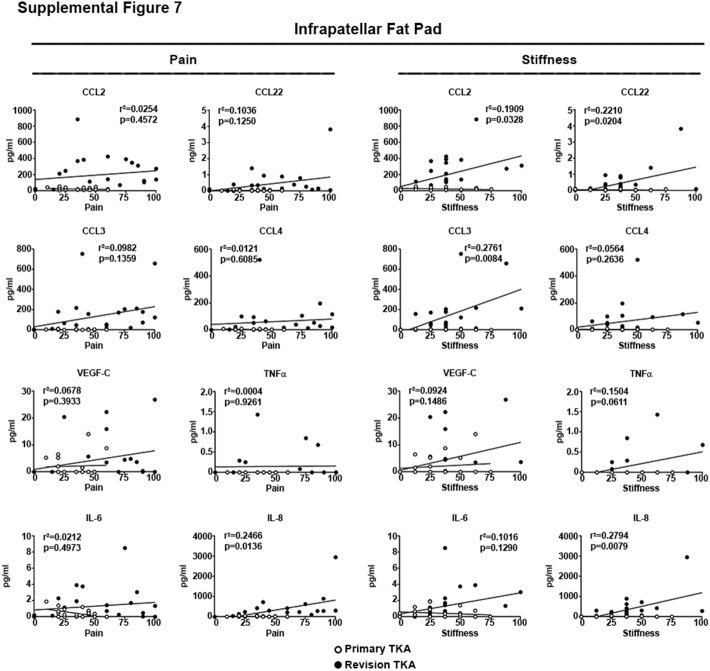
Pain and stiffness are poorly correlated to inflammatory markers in infrapatellar fat pad. Pain and stiffness, as determined by Western Ontario and McMaster Universities Osteoarthritis Index scores, were plotted against expression levels of several markers that were significantly elevated in patients undergoing revision total knee arthroplasty (TKA). Data are presented for patients undergoing primary and revision TKA, and correlations were determined by linear regression analysis. *n* = 20 primary TKA group; *n* = 24 revision TKA group. TNF, tumor necrosis factor; VEGF, vascular endothelial growth factor.

**Supplemental Figure S8:**
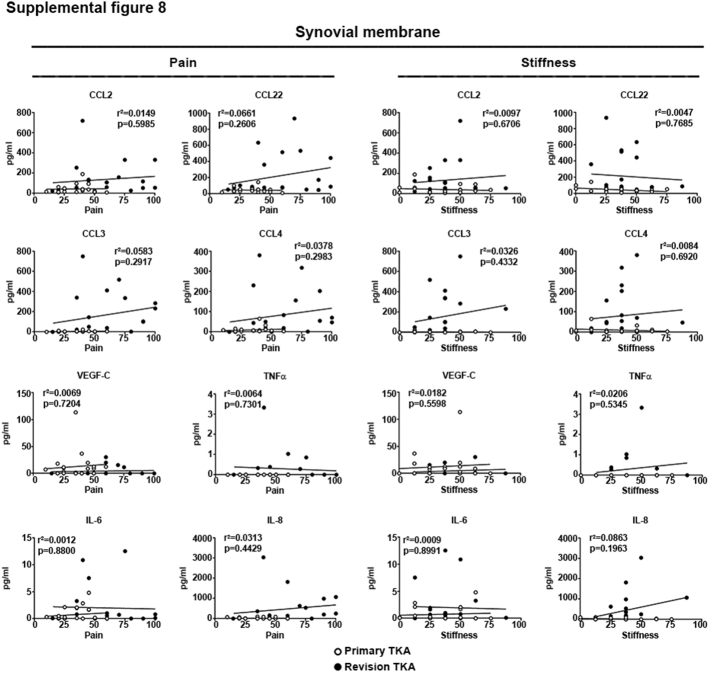
Pain and stiffness are poorly correlated to inflammatory markers in synovial membrane. Pain and stiffness, as determined by Western Ontario and McMaster Universities Osteoarthritis Index scores, were plotted against expression levels of several markers that were significantly elevated in patients undergoing revision total knee arthroplasty (TKA). Data are presented for patients undergoing primary and revision TKA, and correlations were determined by linear regression analysis. *n* = 20 primary TKA group; *n* = 21 revision TKA group. TNF, tumor necrosis factor; VEGF, vascular endothelial growth factor.

Because degree of pain and stiffness are clinical measures of success of the post-TKA joint and patient quality of life,^25^ the degree to which these clinical outcomes correlated with inflammatory markers in the joint was determined. Western Ontario and McMaster Universities Osteoarthritis Index (WOMAC) scores for pain and stiffness were obtained from the Newcastle Freeman Joint Registry for patients consented to this study and correlated to levels of CCL2 and other markers significantly elevated in post-TKA patients. A significant correlation between CCL2 levels in synovial fluid and WOMAC pain scores was observed, but no correlation with WOMAC stiffness scores was found (Figure 7). A significant positive correlation was also seen with vascular endothelial growth factor-C and other markers elevated after TKA, such as IL-8, CCL22, CCL3, and tumor necrosis factor-α; these markers also demonstrated a nonstatistically significant correlation with WOMAC pain scores. Again, there was little to no correlation between any of the markers and WOMAC stiffness scores. Interestingly, levels of CCL2, IL-8, CCL22, and CCL3 in the infrapatellar fat pad were significantly correlated with WOMAC stiffness scores but not with WOMAC pain scores (Supplemental Figure S7). Levels of marker expression did not significantly correlate with either WOMAC pain or stiffness scores in synovial membrane (Supplemental Figure S8).

We conclude that inflammatory markers in the post-TKA synovial fluid correlate with pain and that CCL2, a factor produced by inflammatory fibroblasts in the fibrotic joint, is sufficient to predict pain and identify patients requiring revision TKA surgery.

## Discussion

Modern lower limb arthroplasty results in pain relief and satisfactory outcomes in >80% of patients. However, as many as 20% of patients express post-surgical dissatisfaction because of persistent pain, swelling, loss of movement, and reduced functional ability in the absence of joint instability or infection.^25^ The epidemiology and etiology of this postoperative state remain poorly understood, in particular with respect to pain, yet the symptoms are often sufficiently severe to necessitate early revision surgery, within 5 years of the primary operation. The aim of this study was to determine the nature of the inflammatory process occurring in joints requiring revision surgery and to interrogate the potential role of the fibroblast to contribute to stress-induced inflammatory exacerbations and pain in the post-TKA knee.

Extensive fibrotic remodeling is found to be common to revised TKA joints and is characterized by the accumulation of fibrotic extracellular matrix into the infrapatellar fat pad and synovial membrane.^22^ The fibrotic matrix of the TKA joint is also characterized by the persistence of high numbers of α-smooth muscle actin– and IL-1R1–positive fibroblasts.^19^, ^35^ Herein, we discovered that the fibrotic post-TKA knee is characterized by a chronic inflammatory signature and identify IL-1R1–positive fibroblasts as a potential source of proinflammatory mediators that promote monocyte recruitment and pain in the TKA joint. This occurs via a paracrine pathway in which soluble IL-1α stimulates NF-κB–dependent expression of CCL2. This illuminates a new disease model in which nonresolving fibrosis becomes a driver of inflammation in response to the sterile innate immune triggers (IL-1α). This model has potential relevance to the pathophysiology of all active fibrotic tissues and may help explain the poorly understood association of fibrosis with inflammatory flares and chronic pain in osteoarthritic (OA) joints. We further propose that targeting the fibroblast in the setting of the fibrotic joint offers a novel and rationale approach for suppressing inflammation and pain (ie, worthy of further investigation).

Our key findings are as follows: i) the synovial fluid and infrapatellar fat pad of painful TKA joints displayed elevated expression of multiple inflammatory proteins, including IL-1α and CCL2; ii) bioinformatics analyses of soluble protein expression in the inflamed joint indicated convergence on NF-κB signaling, indicative of a state of chronic unresolved inflammation; iii) fibroblasts in the fibrotic infrapatellar fat pad express IL-1R1 and IL-6 *in vivo* and in response to IL-1α, polarized toward a highly inflammatory phenotype characterized by induction of CCL2 *in vitro*; and iv) CCL2 levels in the joint closely correlated with the degree of pain. From a translational perspective, the main strength of this study is that it identified the association of IL-1α/NF-κB/CCL2 inflammatory signaling in fibroblasts as a driver of inflammation and pain using directly relevant human TKA tissues. Furthermore, this novel pathway offers multiple points for therapeutic intervention using drugs that are safe and clinically effective in humans. A limitation of the study is the limited information available on the revision patients referred into our unit for specialist treatment. Specifically, we do not know whether all of the revision patients had their infrapatellar fat pad excised during their primary surgery and the extent of the synovial resection initially performed. Fat pad resection has been associated with increased anterior knee pain and patella baja, which is suggestive of increased intra-articular scarring.^40^, ^41^

Traditionally, fibroblasts are considered as structural cells that, in response to tissue damage, adopt an activated fibroblast state and promote wound healing and fibrogenesis via their secretion of fibrous collagens and inhibitors of collagenases.^4^ Less well recognized is that these cells exhibit substantial heterogeneity and phenotypic plasticity and can adopt fibroblastic, contractile, or inflammatory states under the influence of cues in their microenvironment.^13^ Polarization of fibroblasts to an inflammatory state is characterized by their acquired expression of IL-6 and IL-8. Studies in fibroblasts derived from multiple tissues (eg, lung, intestine, heart, and skin) indicate that the innate immune trigger IL-1α is a powerful paracrine stimulator of an inflammatory polarization of their phenotype.^12^, ^14^, ^15^, ^17^, ^42^ It was found to be true for fibroblasts derived from the infrapatellar fat pad, such that exposure to pg/mL levels of IL-1α induced ng/mL levels of secreted IL-6, IL-8, and CCL2. More important, IL-1R1 and IL-6 expression was located primarily to fibroblasts in the fibrotic infrapatellar fat pad, whereas soluble IL-1α, IL-6, IL-8, and CCL2 were also elevated and correlated with IL-1α levels in the infrapatellar fat pad of the TKA joint. An important future question will be to identify the cellular source of IL-1α in the joint because this information will reveal the underlying cellular damage/stress mechanisms that trigger the activation of inflammatory fibroblasts of fibrotic tissue. Potential resident sources of IL-1α in the knee include macrophage-like type A and fibroblast-like type B synovial cells.^43^, ^44^

Fibroblasts persisting in the fibrotic TKA joint express IL-1R1, indicating they are primed for polarization to an inflammatory state on exposure to IL-1α. Scarpa et al^42^ reported that fibroblasts isolated from inflammatory bowel disease tissue expressed IL-1R1, as did intestinal subepithelial fibroblasts in the intestine of mice challenged with a low dose of dextran sulfate sodium. More important, after mice had recovered from this mild intestinal injury, a subsequent intrarectal administration of IL-1α induced severe colitis, whereas mice that had not undergone prior dextran sulfate sodium–induced tissue remodeling failed to mount an inflammatory response to IL-1α. The appearance of IL-1R1–positive fibroblasts, therefore, appears to constitute a tissue memory of previous damage that primes the tissue for a robust inflammatory flare when these cells engage IL-1α in the microenvironment. This concept challenges the traditional simple linear model of fibrogenesis in which inflammation is the driver of fibrosis. Instead, we propose a dynamic positive feed-forward process in which inflammatory fibroblasts sense local tissue damage and, via release of CCL2 and other chemokines, promote the recruitment and activation of innate immune cells to drive inflammation; this process constitutes a circulus vitiosus.

CCL2 classically operates as a chemoattractant for monocytes by binding to its receptor CCR2.^45^, ^46^ CCL2 is highly expressed in the synovial fluid of OA joints and knees after trauma and, as observed herein, is a feature of the inflammatory synovium and infrapatellar fat pad of the fibrotic TKA joint.^47^, ^48^, ^49^, ^50^ Although no data directly demonstrating that CCL2 is driving the recruitment of monocytes *in vivo* have been presented, we hypothesize that the elevated levels of CCL2 in the post-TKA knee are likely one of the factors contributing to the recruitment of CD68^+^ cells in a similar manner to what has recently been reported in OA.^51^ An unexpected and novel finding was the close correlation between levels of CCL2 in the synovial fluid of the TKA joint and reported WOMAC pain scores, although no correlation was observed between levels of CCL2 in infrapatellar fat pad and synovial membrane and reported WOMAC pain scores. Although the synovial fluid is unlikely to contain significant numbers of fibroblasts, it is highly likely that fibroblasts present within joint tissues (including the infrapatellar fat pad and synovial membrane as well as other intra-articular or periarticular tissues, such as the joint capsule) are contributing to chronic inflammation in the synovial fluid, as has been previously reported in OA.^52^ In this context, it is intriguing that sensory neurons express CCR2 and, furthermore, CCL2-CCR2 engagement leads to excitation of nociceptive neurons, an event usually associated with the sensation of pain.^53^, ^54^, ^55^, ^56^ In a murine model of OA, genetic deletion of either CCL2 or CCR2 delayed the onset of pain-related behavior.^57^ These observations warrant future studies examining the potential for the use of emerging small-molecule inhibitors of CCR2 as therapies for relieving inflammation and intractable pain in the OA and/or post-TKA joint. Alternatively, a similar outcome may be achieved by the local administration of the IL-1R1 antagonist, anakinra; indeed, there is prior clinical evidence that this approach may be beneficial in TKA.^58^ Because NF-κB signaling appears to be pivotal for IL-1α–induced expression of CCL2 by fibroblasts, there may also be an analgesic benefit to be gained from the local administration of small-molecule inhibitors of NF-κB or its upstream kinase, IKK.^59^, ^60^ Such an approach may also reduce numbers of fibroblasts in the fibrotic tissue because suppression of NF-κB in these cells is associated with the induction of apoptosis.^61^

In summary, this study identified an important role for fibrosis-associated fibroblasts as sensors of cellular stress/damage via engagement of surface-expressed IL-1R1 with the alarmin IL-1α, which leads to secretion of CCL2, a well-established stimulator of monocyte recruitment and nociceptive pain. We hypothesize that IL-1R1–expressing fibroblasts persist in unresolved fibrotic tissue and prime the tissue for inflammatory episodes in response to local cell stress/damage. Finally, the inflamed post-TKA joint is an important new human disease model with which to further illuminate the complex cross talk between fibrosis, inflammation, and pain.
